# 衍生化-气相色谱-三重四极杆质谱法测定泼尼松龙中联氨

**DOI:** 10.3724/SP.J.1123.2021.03002

**Published:** 2021-07-08

**Authors:** Chong QIAN, Mei ZHANG, Shanshan LIU, Xinlei GOU, Wei WANG, Guanghui HU

**Affiliations:** 1.北京市理化分析测试中心, 有机材料检测技术与质量评价北京市重点实验室 北京 100089; 1. Beijing Key Laboratory of Organic Materials Testing Technology & Quality Evaluation Beijing Centre for Physical and Chemical Analysis, Beijing 100089, China; 2.北京市食品安全分析测试工程技术研究中心, 北京 100089; 2. Beijing Engineering Research Center of Food Safety Analysis, Beijing 10089, China

**Keywords:** 气相色谱-三重四极杆质谱, 联氨, 泼尼松龙, gas chromatography-triple quadrupole mass spectrometry (GC-MS/MS), hydrazine, prednisolone

## Abstract

泼尼松龙是一种广泛用于临床治疗的肾上腺糖皮质激素药物,其中联氨的残留会直接影响用药安全,但目前国内外还没有出台相应的法律法规和标准来管控药物中联氨的残留限值。联氨具有强极性和强还原性,理化性质很不稳定,易被氧化,又因缺少发色团,相对分子质量太小,检测起来难度很大,需引入一种衍生化试剂,降低其极性,生成相对分子质量较大且理化性质稳定的衍生产物。该研究通过优化衍生化试剂、色谱-质谱条件、溶剂体系和衍生化条件,建立了衍生化-气相色谱-三重四极杆质谱法(GC-MS/MS)测定泼尼松龙中联氨残留的方法,并进行了方法学验证,结果满意。称取1 g泼尼松龙样品置于10 mL具塞离心管中,加入稀释溶剂(甲醇-二氯甲烷(14:23, v/v))至刻度线,涡旋振荡至样品完全溶解后,吸取100 μL置于进样小瓶中,再加入丙酮900 μL,涡旋振荡混匀,样品在丙酮-稀释溶剂(9:1, v/v)中同时完成稀释和衍生化反应后,再经GC-MS/MS检测分析。该研究的衍生化反应无需在添加冰乙酸和超声条件下进行,也无需再添加其他试剂进行萃取操作,联氨与丙酮可瞬间发生衍生化反应,直接实现泼尼松龙中联氨的快速测定。结果表明,联氨在1~12 μg/L范围内线性关系良好,线性相关系数(*r*^2^)为0.9999;检出限、定量限分别为0.03和0.10 mg/kg;进样精密度(relative standard deviation, RSD)为1.10%。加标回收率和重复性良好,加标水平分别为1、6、12 μg/L时的回收率为96.15%~96.46%,对应的RSD值为1.77%~2.12%。中间精密度良好,不同时间、不同人员在同一台仪器上测定结果的RSD值为1.77%。方法耐用性良好,通过改变色谱条件来研究检测结果受影响程度大小,在原条件、初始柱温±5 ℃、升温速率±2 ℃/min、柱流量±0.1 mL/min的条件下分别对加标6 μg/L的样品溶液中的联氨含量进行检测,检测结果的RSD值为2.58%。应用建立的方法测定泼尼松龙市售标准品和某药企提供的9个不同批次的泼尼松龙样品,均未检出联氨。该方法操作简便、准确可靠、灵敏度高、选择性好,可用于泼尼松龙中联氨的检测。

## Abstract (en)

Prednisolone is an adrenal glucocorticoid drug with immunosuppressive, anti-inflammatory, anti-allergic, and antiviral effects that are widely exploited in clinical treatment. The hydrazine residue to prednisolone directly affects medication safety and threatens the patient’s health. At present, there are no relevant laws, regulations, and standards to control the residual limit of hydrazine in drugs at home or abroad. Therefore, a simple, rapid, accurate, reliable, sensitive, and selective method is urgently needed for the determination of trace hydrazine in prednisolone. Hydrazine has strong polarity and reductivity, with unstable physical and chemical properties, thus being easily oxidized. In addition, because of the lack of chromophores and low molecular weight, the detection of hydrazine is very difficult. Therefore, a derivative reagent should be introduced to reduce its polarity and generate a derivative product with a high molecular weight as well as stable physical and chemical properties. Acetone, as a common laboratory reagent, is inexpensive and can rapidly react with hydrazine; therefore, it is an ideal derivative reagent for the determination of hydrazine. In this study, a method based on precolumn derivatization with gas chromatography-triple quadrupole mass spectrometry (GC-MS/MS) was developed for the determination of hydrazine in prednisolone by optimizing the derivatization reagent, GC and MS conditions, solvent system, and derivatization conditions. Method validation was then carried out using the established method, and the results were satisfactory. In this study, 1 g of prednisolone sample was weighed and placed in a 10 mL centrifuge tube with a plug; then, a methanol-dichloromethane dilution solvent (14:23, v/v) was added to the scale line, and the sample was vortexed until completely dissolved. About 100 μL of the test solution prepared above was pipetted into the sample vial, followed by the addition of 900 μL acetone. The resulting solution was vortexed and mixed well. The sample was diluted and derivatized simultaneously in acetone solution, acetone/methanol-dichloromethane dilution solvent (9:1, v/v), and then detected and analyzed by GC-MS/MS. In this study, the derivatization reaction between hydrazine and acetone did not require the addition of acetic acid and ultrasound conditions, or the use of other reagents for the extraction operation. The reaction was instantaneous, and rapid determination of hydrazine in prednisolone could be achieved. The standard curve was obtained with a good correlation coefficient (*r*^2^=0.9999) in the range 1-12 μg/L. The limits of detection and quantitation were 0.03 mg/kg and 0.10 mg/kg, respectively. The relative standard deviation (RSD) of injection precision was 1.10%. The recoveries and repeatability were good; the recoveries of low-, medium-, and high-concentration spiked samples were 96.15%-96.46% at spiked concentrations of 1, 6, and 12 μg/L, respectively, and the corresponding RSDs were 1.77%-2.12%. The intermediate precision was good, and the RSD of the determination results obtained on the same instrument by different laboratory technicians at different times was 1.77%. The durability was good, and the degree of influence of the detection results was studied by changing the chromatographic conditions. Under the original condition or conditions with initial column temperature ±5 ℃, heating rate ±2 ℃/min, or column flow rate ±0.1 mL/min, the hydrazine content in the sample solution at a spiked concentration of 6 μg/L was detected, and the RSD of the detection results was 2.58%. The established method was applied to detect hydrazine in a prednisolone standard substance procured from the market and nine batches of prednisolone samples provided by a pharmaceutical company. No hydrazine was detected in any of these samples. The established method is simple, reliable, highly sensitive, and highly selective, and it can be applied for the detection of hydrazine in prednisolone.

联氨(N_2_H_4_)是一种无色、油状、易燃的液体,通常以水合联氨(N_2_H_4_·H_2_O)的形态存在,是一种重要的化学试剂,被广泛应用于工业、农业和军事等领域,在医药、农药、塑料、染料和火箭燃料等行业发挥着重要作用^[1]^。联氨具有一种类似氨的刺鼻性气味,是腐蚀性极强的强碱,对人体危害较大,是一种刺激性强烈的皮肤致敏物,会损害人体的肝脏、肺、肾脏、血液和中枢神经系统,国际化学安全项目及综合风险信息系统根据该系统的证据特征权重给予联氨B2的分级(可能的人类致癌物质)^[2]^。泼尼松龙(prednisolone)化学名称为11*β*,17*α*,21-三羟基孕甾-1,4-二烯-3,20-二酮,是一种广泛用于临床治疗的肾上腺糖皮质激素药物,具有免疫抑制、抗炎、抗过敏和抗病毒等功效^[3]^。盐酸氨基脲是一种重要的化工中间体,作为医药原料可用于制取硝基呋喃类等药物,是泼尼松龙合成的重要原料,而水合联氨又是盐酸氨基脲合成的原料,所以在泼尼松龙的合成过程中,盐酸氨基脲的使用很有可能会引入联氨的残留^[4]^。根据欧洲药事管理局和美国食品药品管理局的法规要求,基因毒性杂质的毒理学关注阈值(threshold of toxicological concern, TTC)限度为1.5 μg/d,泼尼松龙每天最高服用剂量为250 mg,因此泼尼松龙中联氨的可接受限度为6 μg/g。一般药企在生产泼尼松龙时,为了安全起见,当联氨的残留量低于可接受限度的10%时,才可以确定泼尼松龙产品是安全的,所以本研究将泼尼松龙中联氨的限度定为0.6 μg/g。泼尼松龙中联氨的残留会直接影响用药安全,威胁人们的身体健康。目前,国内外还没有出台相应的法律法规和标准来管控药物中联氨的残留限值^[5]^。所以,建立一种简便快捷、准确可靠、灵敏度高、选择性好的泼尼松龙中痕量联氨的测定方法,对保障临床用药安全具有重要意义^[6]^。

联氨具有强极性和强还原性,其理化性质不稳定,很容易被氧化,在空气中易吸湿冒烟,又因缺少发色团,相对分子质量太小,检测起来难度很大。目前联氨常见的检测方法有分光光度法^[7]^、气相色谱法(gas chromatography, GC)^[8,9]^、气相色谱-质谱法(gas chromatography-mass spectrometry, GC-MS)^[10,11]^、液相色谱法(liquid chromatography, LC)^[12,13]^、液相色谱-串联质谱法(liquid chromatography-tandem mass spectrometry, LC-MS/MS)^[14]^、离子色谱法(ion chromatography, IC)^[15]^、荧光分析法^[16]^等。上述方法中,分光光度法灵敏度太低,不能满足低检出限的要求;GC、LC、IC的基质干扰大,专属性差,复杂基质样品中的其他组分易干扰其检测分析;GC-MS的基质效应较大,重复性不好,累积进样后检测结果偏差较大;LC-MS/MS对实验室条件要求较高,仪器昂贵,检测成本高,不利于检测方法的普及;荧光分析法需要构建特定的荧光探针,操作繁琐。相比较而言,气相色谱-三重四极杆质谱法(gas chromatography-triple quadrupole mass spectrometry, GC-MS/MS)具有检测成本低、操作简便、灵敏度高、专属性好、基质干扰小、基质效应低等优点^[17]^,目前关于药物中联氨的GC-MS/MS检测方法报道较少,缺乏相关研究。周延生等^[18]^、侯勤勤等^[19]^采用丙酮溶液(每毫升丙酮中加入1 μL冰乙酸)作为萃取剂和衍生化试剂,分别对药物和土壤中的联氨进行衍生和超声提取,再使用GC氮磷检测器(nitrogen phosphorus detector, NPD)对联氨进行测定。该方法需在添加冰乙酸和超声条件下进行,样品、试剂用量大,实验操作和NPD检测器维护起来繁琐费时。此外,由于样品处于未溶解状态,会存在联氨提取和衍生不完全的可能。因为泼尼松龙不能溶于丙酮,本研究先采用稀释溶剂甲醇-二氯甲烷(14:23, v/v)溶解泼尼松龙,再在丙酮-稀释溶剂(9:1, v/v)中进行稀释、衍生化,联氨与丙酮的衍生化反应无需在添加冰乙酸和超声条件下进行,也无需再添加其他试剂进行萃取操作,联氨与丙酮可瞬间发生衍生化反应,从而直接实现泼尼松龙中联氨的快速测定。本文建立了衍生化-GC-MS/MS检测泼尼松龙中联氨的方法,并进行了相关的方法学验证,取得了满意的结果,为泼尼松龙及其他药物中联氨的检测和监控提供了科学依据和技术支持。

## 1 实验部分

### 1.1 仪器与试剂

GCMS-TQ8040气相色谱-三重四极杆质谱仪,GCMSsolution 4.30工作站(日本Shimadzu公司); XPE105电子天平(瑞士Mettler Toledo公司); Vortex-Genie 2涡旋振荡器(美国Scientific Industries公司)。

甲醇、二氯甲烷、丙酮(色谱纯,美国Thermo Fisher Scientific公司);冰乙酸(分析纯,北京化工厂);联氨标准溶液(1000 mg/L,国家有色金属及电子材料分析测试中心国标(北京)检验认证有限公司);泼尼松龙标准品(纯度为99.74%,德国Dr. Ehrenstorfer公司);泼尼松龙样品(某制药公司提供,用于方法学验证试验)。

### 1.2 稀释溶剂的配制

准确量取甲醇140 mL、二氯甲烷230 mL置于同一试剂瓶中,摇匀,配制得甲醇-二氯甲烷(14:23, v/v)混合溶液,作为稀释溶剂,在4 ℃下保存。

### 1.3 空白溶液的配制

吸取900 μL丙酮置于进样小瓶中,再加入100 μL稀释溶剂,涡旋振荡混匀,作为空白溶剂。

### 1.4 标准工作溶液的配制

用丙酮将1000 mg/L的联氨标准溶液原液逐级梯度稀释至10、20、40、60、100、120 μg/L,分别吸取上述联氨标准溶液各100 μL置于不同的进样小瓶中,再分别加入稀释溶剂100 μL和丙酮800 μL,涡旋振荡混匀,即配制得质量浓度分别为1、2、4、6、10、12 μg/L的标准工作溶液。所有标准溶液在4 ℃下保存。

### 1.5 样品处理

称取泼尼松龙样品1 g置于10 mL具塞离心管中,加入稀释溶剂至刻度线,涡旋振荡至样品完全溶解,配制得100 g/L的样品测试液。吸取上述配制的样品测试液100 μL置于进样小瓶中,再加入丙酮900 μL,涡旋振荡混匀,即配制得10 g/L的样品测试液,作为供试品溶液。

### 1.6 GC-MS/MS分析条件

GC条件:VF-5 MS毛细管色谱柱(30 m×0.25 mm×0.25 μm);进样口温度250 ℃;载气为高纯He(纯度>99.999%);分流进样,分流比5:1;进样量1.0 μL;恒线速度控制模式;柱流量1.0 mL/min;升温程序为,初始柱温50 ℃保持1 min, 20 ℃/min升至150 ℃, 30 ℃/min升至280 ℃,保持5 min。

MS/MS条件:电子轰击离子源(EI);电子能量70 eV;离子源温度230 ℃;接口温度280 ℃;溶剂延迟时间2 min;多重反应监测模式(multiple reaction monitoring, MRM);联氨衍生物的保留时间为3.573 min;定量离子对为*m/z* 112.00>97.10,对应的碰撞能为6 V;定性离子对为*m/z* 112.00>56.10、*m/z* 112.00>70.10,对应的碰撞能为18、9 V。

## 2 结果与讨论

### 2.1 衍生试剂的选择

联氨强极性的氨基基团会与毛细管色谱柱的固定相发生作用,导致色谱峰展宽严重,同时还会引起严重的柱流失,干扰检测。联氨的相对分子质量小,如果直接使用GC-MS/MS进行检测,易被基质和系统中的其他碎片离子干扰,同时还会因为生成的母离子相对分子质量太小,无法二次打碎生成相应的子离子,无法进行GC-MS/MS分析。此外,联氨还具有强还原性,理化性质不稳定,在检测过程中很容易发生氧化反应,使检测结果失真,甚至还会出现假阴性的结果。所以,在联氨的GC-MS/MS检测过程中需引入一种衍生试剂,降低其极性,生成相对分子质量较大且理化性质稳定的衍生产物。目前文献报道的衍生化试剂有丙酮^[18,19]^、苯甲醛^[2]^、对二甲氨基苯甲醛^[7]^、氯甲酸乙酯^[8]^、糠醛^[9]^、邻苯二甲醛^[10]^、2-硝基苯甲醛^[11]^、三氟乙酰基丙酮^[17]^等,其中,由于丙酮为实验室常用试剂,且价格低廉,有利于本方法的普及和推广,因此本研究选择丙酮作为联氨衍生化试剂,联氨与丙酮的衍生反应方程式如图1所示。

**图 1:**
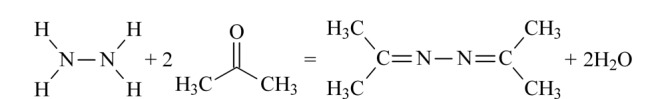
联氨与丙酮的衍生化反应

### 2.2 色谱-质谱条件的优化

用丙酮将质量浓度为1000 mg/L的联氨标准溶液原液稀释至10 mg/L,然后在*m/z* 60~115范围内进行全扫描(scan)分析,依次对丙酮和联氨标准溶液进行检测。由于丙酮和联氨衍生物(1,2-二(丙烷-2-亚烷基)肼)的相对分子质量分别为58和112,所以在该条件下,不仅可以完全消除丙酮以及基质中*m/z*大于115的碎片离子的干扰,同时还能满足联氨衍生物的scan分析。图2为丙酮中干扰杂质和联氨衍生物总离子流图的叠加图,丙酮中有干扰杂质与联氨衍生物的保留时间一致,干扰联氨的检测分析。图3为丙酮杂质和联氨衍生物的质谱图,比较分析发现*m/z* 101的碎片离子同时存在于丙酮杂质和联氨衍生物的质谱图中,且强度相近,而*m/z* 97、*m/z* 112的碎片离子只存在于联氨衍生物质谱图中,表明*m/z* 101为丙酮基质中干扰杂质的碎片离子,而*m/z* 97、*m/z* 112为联氨衍生物的特征碎片离子。联氨衍生物的一级裂解规律如图4所示。

**图 2:**
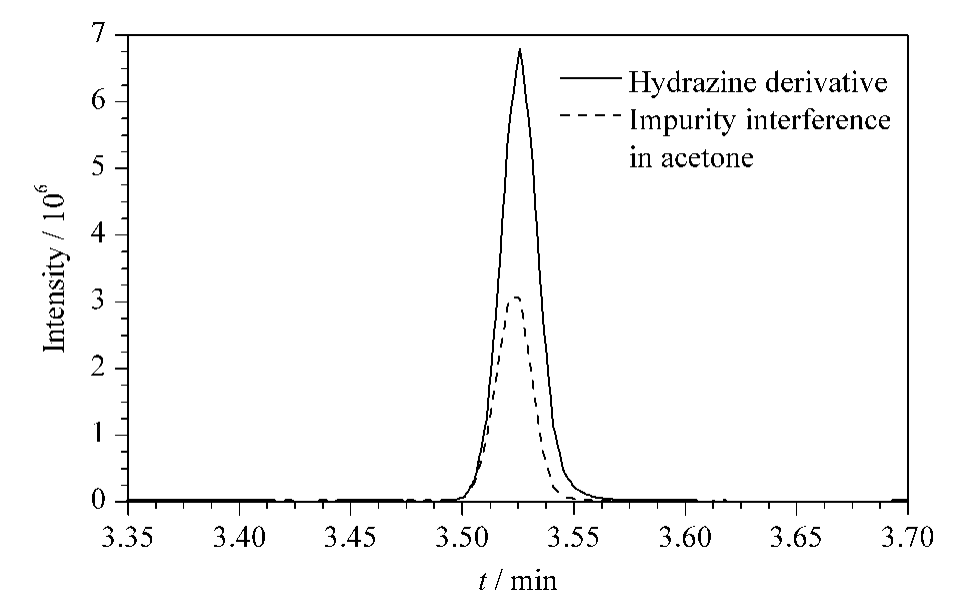
丙酮、联氨衍生物总离子流图的叠加图

**图 3:**
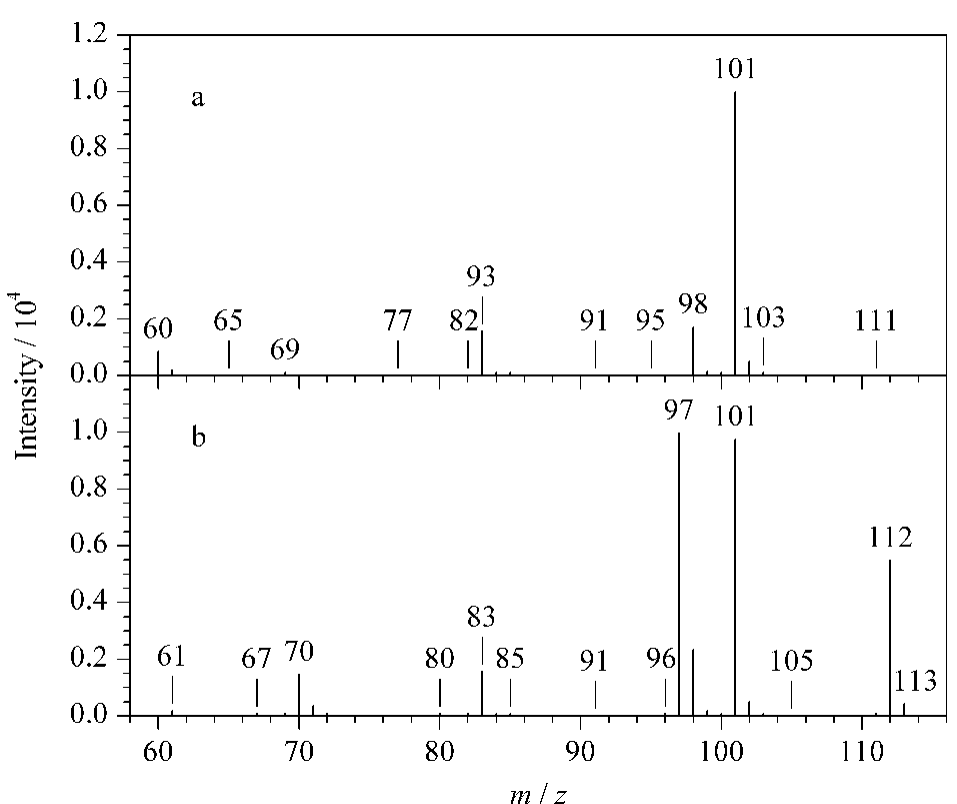
(a)丙酮中干扰杂质和(b)联氨衍生物的质谱图

**图 4:**
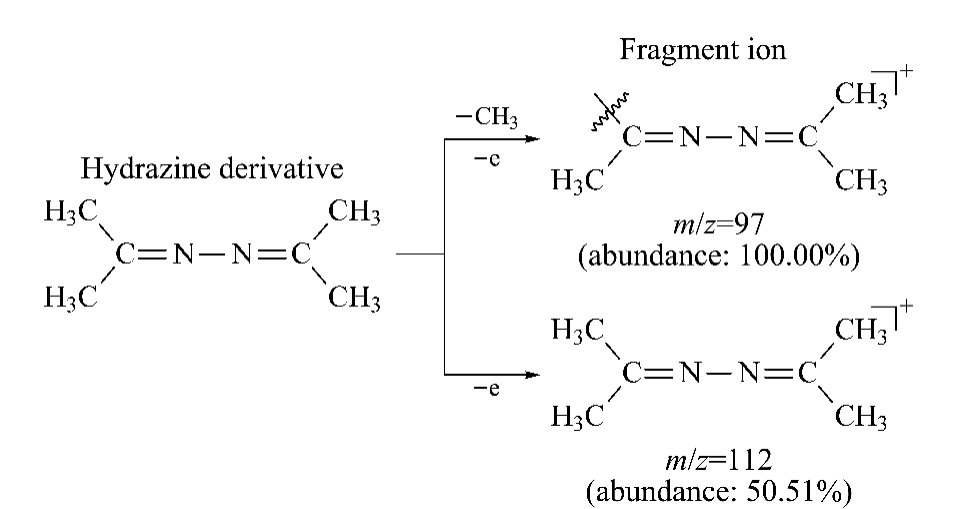
联氨衍生物的一级裂解规律

选取丰度高、质荷比大的特征碎片离子*m/z* 97、*m/z* 112作为母离子(一级碎片离子),进行二级质谱分析,对其进行碰撞能优化,对子离子(二级碎片离子)进行优化选择,选择丰度较高、质荷比较大、基质干扰较小的离子对进行定性、定量分析。表1为联氨衍生物的离子对优化结果,由于丙酮中存在干扰联氨分析的离子对*m/z* 97.00>56.10(见图5),所以本方法舍弃该离子对,选取*m/z* 112.00>97.10(定量离子对),以及*m/z* 112.00>56.10、*m/z* 112.00>70.10(定性离子对)对联氨进行检测分析。图6为该条件下空白溶液和供试品溶液的选择离子流图,表明空白溶液、供试品溶液中其他组分不会干扰联氨的检测分析,该方法专属性良好。再对分流比、升温程序、柱流量、进样口温度、接口温度等色谱参数进行优化调整,使联氨衍生物的响应和峰形均达到最佳,缩短方法检测程序的时间,提高检测效率。联氨衍生物的二级裂解规律如图7所示。

**表 1:** 联氨衍生物的离子对

No.	Ion pair (m/z)	Collision energy/V	Abundance/%
1	112.00>97.10	6	100.00
2	112.00>56.10	18	27.68
3	97.00>56.10	9	25.83
4	112.00>70.10	9	8.03

**图 5:**
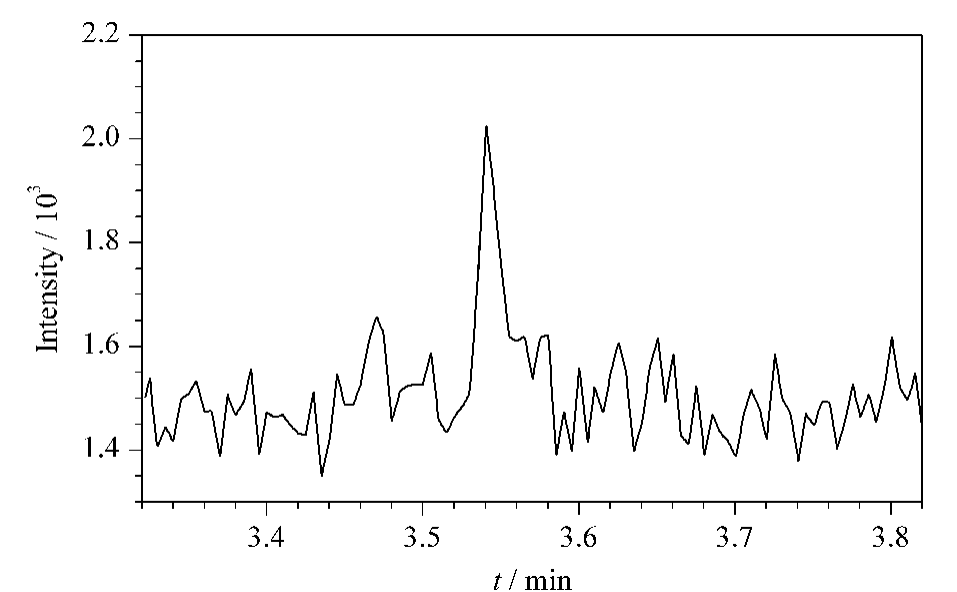
丙酮的选择离子流图

**图 6:**
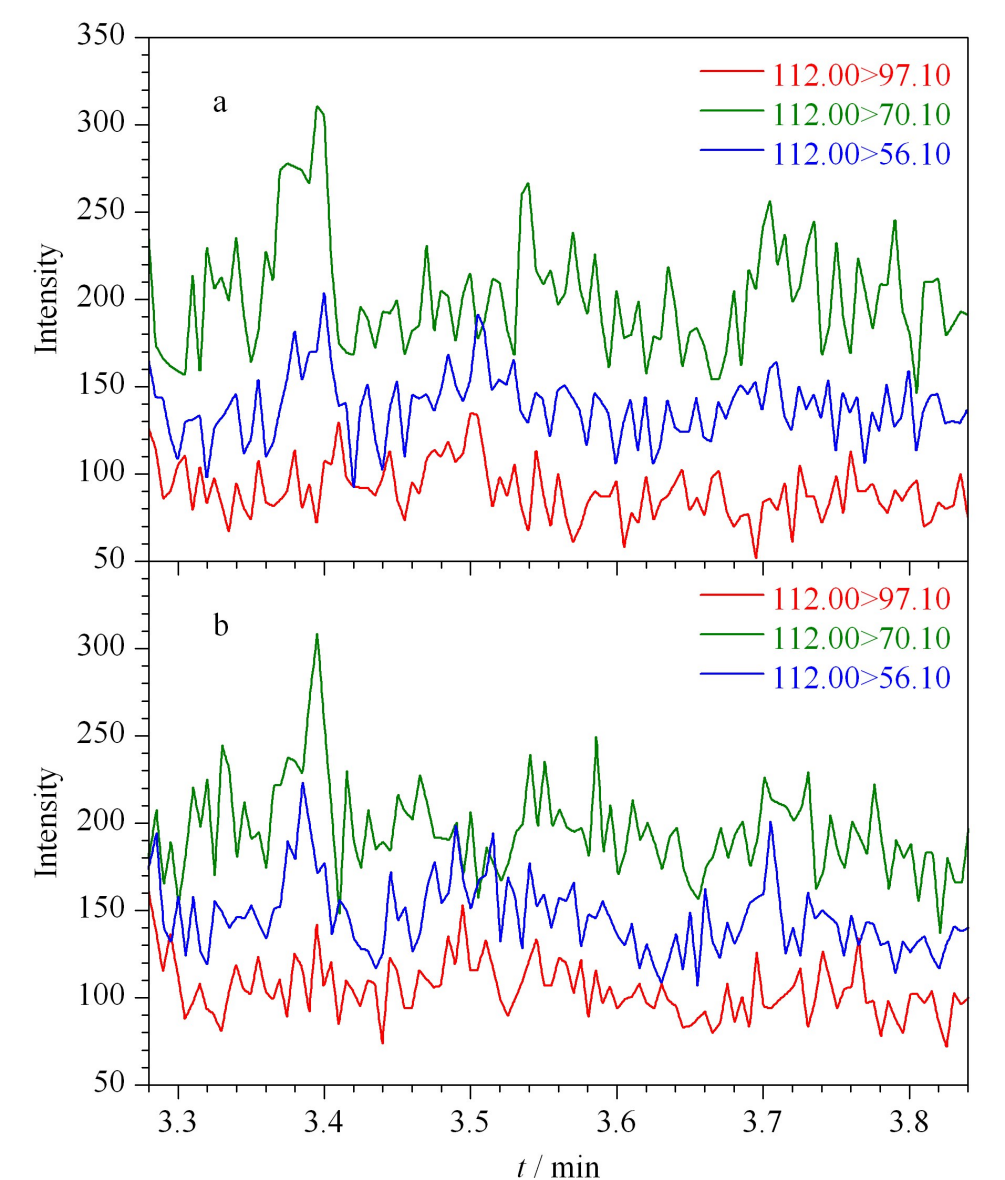
(a)空白溶液和(b)供试品溶液的选择离子流图

**图 7:**
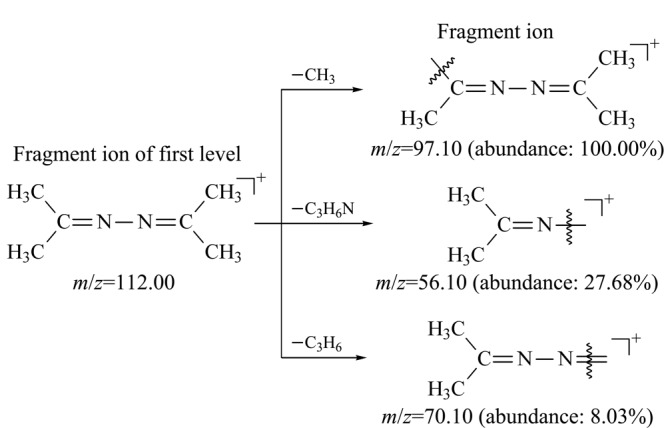
联氨衍生物的二级裂解规律

### 2.3 溶剂体系的优化

平行称取10份泼尼松龙样品100 mg于离心管中,分别加入水、甲醇、乙腈、二氯甲烷、丙酮、*N*,*N*-二甲基甲酰胺、二甲基亚砜、正己烷、乙酸乙酯、甲苯各1 mL,充分涡旋振荡,考察样品的溶解情况。发现除*N*,*N*-二甲基甲酰胺、二甲基亚砜能完全溶解,乙腈能部分溶解外,其他溶剂的溶解效果均不理想。同时对联氨衍生物在*N*,*N*-二甲基甲酰胺、二甲基亚砜、乙腈体系中的响应情况进行考察,发现联氨衍生物在乙腈体系中的色谱峰严重前延,在*N*,*N*-二甲基甲酰胺、二甲基亚砜体系中响应很低,且峰形不佳。

上述实验结果表明,使用单一溶剂难以同时实现样品溶解和联氨衍生物响应良好,因此,本研究尝试使用混合溶剂体系来解决样品的溶解和目标物质响应的问题。研究发现,采用甲醇-二氯甲烷混合溶剂时,泼尼松龙的溶解有很大改善。甲醇、二氯甲烷体积比在11:29至22:18范围内时,100 mg泼尼松龙能完全溶于1 mL甲醇-二氯甲烷混合溶剂;当甲醇、二氯甲烷体积比为14:23时,泼尼松龙的溶解度最好。综上,本研究先采用甲醇-二氯甲烷(14:23, v/v)作为稀释溶剂来溶解泼尼松龙样品,再用丙酮将其稀释10倍,即最终的溶剂体系为丙酮-稀释溶剂(9:1, v/v)。在该溶剂体系下,联氨标准溶液(6.00 μg/L)的选择离子流图如图8所示,联氨衍生物色谱峰响应、峰形均良好。

**图 8:**
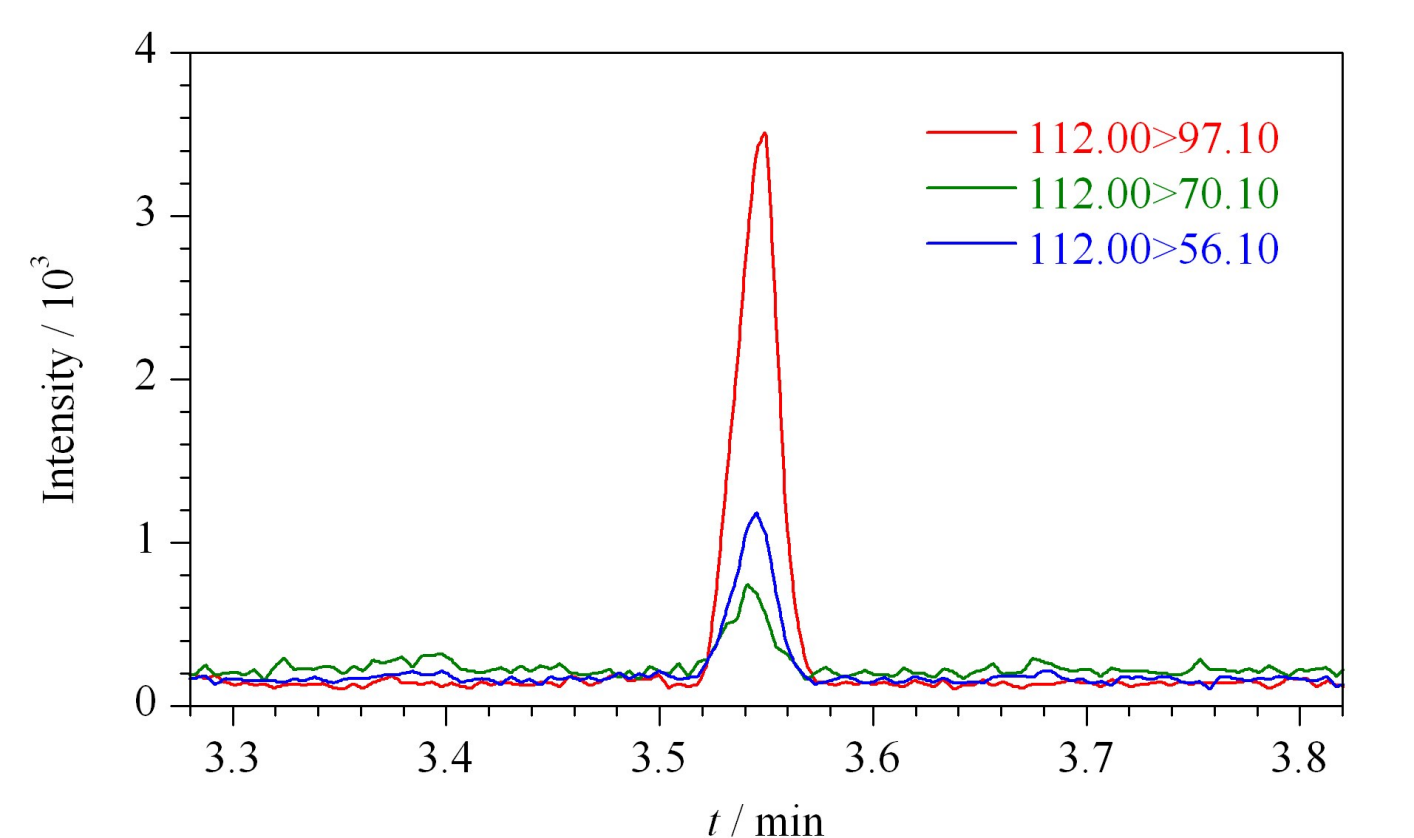
6.00 μg/L联氨标准溶液的选择离子流图

### 2.4 衍生化条件的优化

文献^[18,19]^报道,当采用丙酮作为衍生化试剂检测联氨时,需添加冰乙酸和在超声条件下进行衍生化反应。本研究从超声时间和冰乙酸添加量两方面对衍生化条件进行优化。首先配制冰乙酸含量为1 mL/L的丙酮溶液,再用该溶液按1.4节方法配制质量浓度为6.00 μg/L的联氨标准溶液,将该溶液平行分装成8份置于进样小瓶中,分别超声处理0、1、2、5、10、15、30、60 min后,分析联氨标准溶液定量离子对*m/z* 112.00>97.10峰面积的变化趋势(见图9a)。超声时间在0~60 min范围内时,联氨标准溶液的峰面积稳定,联氨的衍生反应与超声时间的长短没有关系。分别配制冰乙酸质量浓度为0、1、2、5、10、25 mL/L的丙酮溶液,再按1.4节方法分别配制6.00 μg/L的联氨标准溶液,不同冰乙酸浓度体系下,联氨标准溶液定量离子对*m/z* 112.00>97.10峰面积的变化趋势如图9b所示。当冰乙酸质量浓度为0~5 mL/L时,联氨标准溶液峰面积稳定;当冰乙酸浓度为5~25 mL/L时,随着冰乙酸浓度的增加,联氨标准溶液峰面积有逐渐减小的趋势,表明联氨的衍生化反应不需要在添加冰乙酸的条件下进行。上述结果表明,联氨与丙酮混合后可直接发生衍生化反应,该反应不需要添加冰乙酸,也不需要在超声条件下进行。

**图 9:**
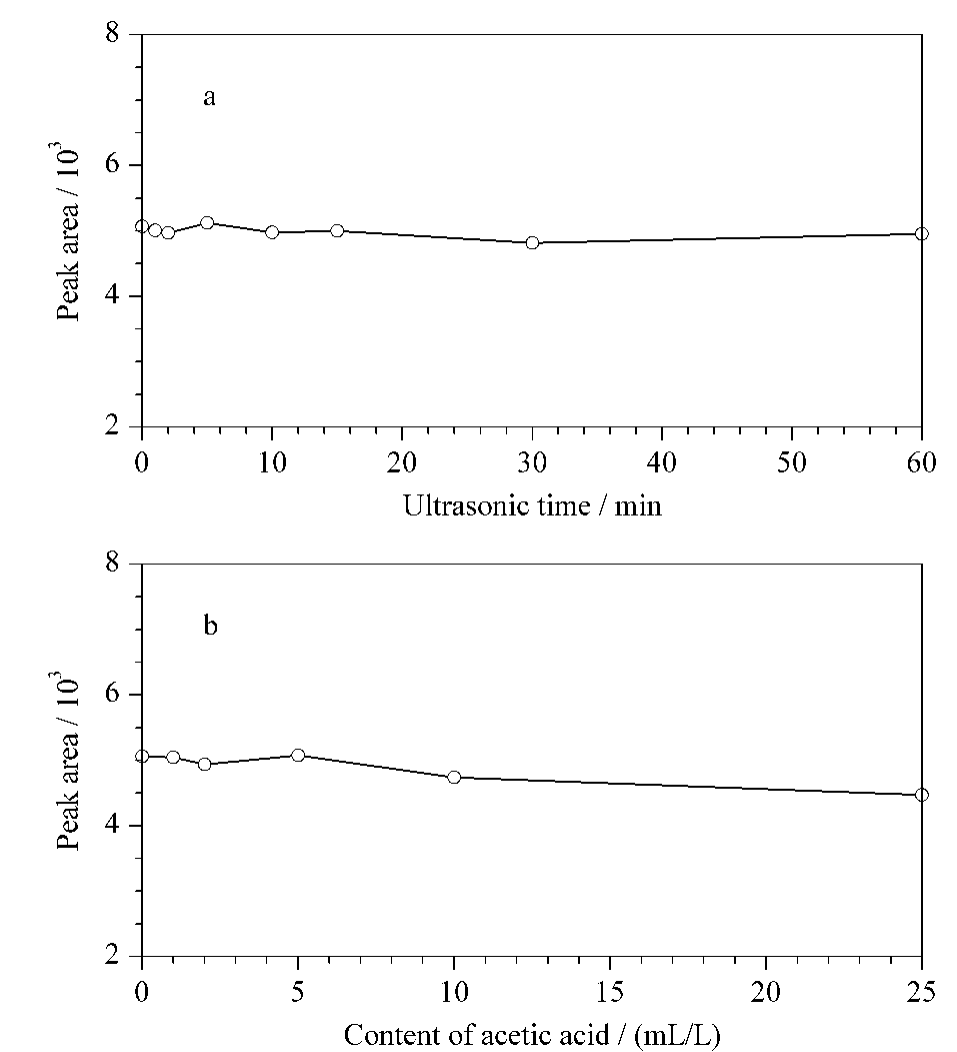
(a)不同超声时间和(b)乙酸浓度下的联氨标准溶液的峰面积(*m/z* 112.00>97.10)

### 2.5 方法检出限、定量限和线性范围

用优化好的方法依次分析按照1.4节配制的标准工作溶液,以联氨衍生物定量离子对*m/z* 112.00>97.10的峰面积(*y*)和联氨质量浓度(*x*, μg/L)进行线性回归,联氨在1~12 μg/L范围内线性关系良好,得到线性回归方程*y*=811.41*x*+56.43和线性相关系数*r*^2^=0.9999。分别以10倍信噪比(*S/N*)和3倍*S/N*确定联氨的方法定量限和检出限为0.10和0.03 mg/kg。结果表明本方法适用于泼尼松龙中痕量联氨的检测分析。

### 2.6 进样精密度

按1.4节配制6.00 μg/L的联氨标准溶液,连续测定6次,以联氨衍生物定量离子对*m/z* 112.00>97.10峰面积的RSD值考察方法的进样精密度。6次峰面积的RSD值为1.10%,表明本方法进样精密度良好。

### 2.7 加标回收率和重复性

对泼尼松龙市售标准品和某药企提供的9个不同批次的泼尼松龙样品进行检测分析,均未检出联氨残留。配制加标质量浓度分别为1、6、12 μg/L的低、中、高水平空白加标样品溶液,每个加标水平平行制备6份样品进行检测。结果如表2所示,不同加标水平下,联氨的平均回收率为96.15%~96.46%, RSD值为1.77%~2.12%,能满足联氨的检测要求。

**表 2:** 联氨的加标回收率和重复性(*n*=6)

Analyte	1 μg/L			6 μg/L			12 μg/L	
	Recovery/ %	RSD/ %		Recovery/ %	RSD/ %		Recovery/ %	RSD/ %
Hydrazine	96.28	2.12		96.15	1.84		96.46	1.77

### 2.8 中间精密度

不同时间、不同人员分别平行配制6份加标水平为6 μg/L的空白加标样品溶液,在同一台仪器上重复测定,12次测定的平均加标回收率为96.23%, RSD值为1.77%,表明本方法的中间精密度良好。

### 2.9 方法的耐用性

本方法通过改变色谱条件来研究检测结果受影响的程度。分别在原条件、初始柱温±5 ℃、升温速率±2 ℃/min、柱流量±0.1 mL的条件下检测6.00 μg/L的联氨标准溶液和6 μg/L的空白加标样品溶液,外标法单点计算加标回收样品溶液中联氨的含量,结果如表3所示。联氨检测结果的RSD值为2.58%,表明本方法耐用性良好,色谱条件的适当改变不会对检测结果造成太大影响。

**表 3:** 联氨检测方法的耐用性

Analyte	C/(μg/L)									RSD/ %
	Original condition	Δ(initial column temperature)/℃			Δ(heating rate)/(℃/min)			Δ(column flow rate)/(mL/min)		
		+5	-5		+2	-2		+0.1	-0.1	
Hydrazine	5.84	5.63	5.89		5.91	5.72		5.78	5.50	2.58

## 3 结论

本研究建立了衍生化-GC-MS/MS测定泼尼松龙中联氨的分析方法。样品先经甲醇-二氯甲烷(14:23, v/v)溶解,再经丙酮稀释、衍生后,直接上机检测分析。该方法操作简便、专属性强、检出限低、准确度高、基质干扰小、基质效应低、耐用性好,能够满足泼尼松龙中联氨的检测要求,为泼尼松龙及其他药物中联氨的检测及监控提供了科学依据和技术支持。
